# Performance Characterization and Composition Design Using Machine Learning and Optimal Technology for Slag–Desulfurization Gypsum-Based Alkali-Activated Materials

**DOI:** 10.3390/ma17143540

**Published:** 2024-07-17

**Authors:** Xinyi Liu, Hao Liu, Zhiqing Wang, Xiaoyu Zang, Jiaolong Ren, Hongbo Zhao

**Affiliations:** School of Civil Engineering and Geomatics, Shandong University of Technology, Zibo 255000, China; 22507020003@stumail.sdut.edu.cn (X.L.); 23507030872@stumail.sdut.edu.cn (H.L.); 22507020019@stumail.sdut.edu.cn (X.Z.);

**Keywords:** alkali-activated materials, performance characterization, composition design, machine learning, simplicial homology global optimization

## Abstract

Fly ash–slag-based alkali-activated materials have excellent mechanical performance and a low carbon footprint, and they have emerged as a promising alternative to Portland cement. Therefore, replacing traditional Portland cement with slag–desulfurization gypsum-based alkali-activated materials will help to make better use of the waste, protect the environment, and improve the materials’ performance. In order to better understand it and thus better use it in engineering, it needs to be characterized for performance and compositional design. This study developed a novel framework for performance characterization and composition design by combining Categorical Gradient Boosting (CatBoost), simplicial homology global optimization (SHGO), and laboratory tests. The CatBoost characterization model was evaluated and discussed based on SHapley Additive exPlanations (SHAPs) and a partial dependence plot (PDP). Through the proposed framework, the optimal composition of the slag–desulfurization gypsum-based alkali-activated materials with the maximum flexural strength and compressive strength at 1, 3, and 7 days is Ca(OH)_2_: 3.1%, fly ash: 2.6%, DG: 0.53%, alkali: 4.3%, modulus: 1.18, and W/G: 0.49. Compared with the material composition obtained from the traditional experiment, the actual flexural strength and compressive strength at 1, 3, and 7 days increased by 26.67%, 6.45%, 9.64%, 41.89%, 9.77%, and 7.18%, respectively. In addition, the results of the optimal composition obtained by laboratory tests are very close to the predictions of the developed framework, which shows that CatBoost characterizes the performance well based on test data. The developed framework provides a reasonable, scientific, and helpful way to characterize the performance and determine the optimal composition for civil materials.

## 1. Introduction

Cement is the most commonly used concrete cementitious material in the world. However, much CO_2_ is emitted during its production. Cement production accounts for 5–7% of global CO_2_ emissions [1,2,3,4]. In order to protect the environment, there is an urgent need for environmentally friendly adhesive materials that replace cement. Alkali-activated materials are one of the most important substitutes for cement due to their low cost, high mechanical strength, fire resistance, and low energy consumption [5]. Meanwhile, they have a smaller carbon footprint than cement [3]. Also, alkali-activated materials have disadvantages such as slower strength growth, having rheological properties that are difficult to evaluate [6], the interference of the type of curing in the mechanical properties [7], and environmental costs. However, if the composition of the alkali-activated material can be reasonably designed, better performance can be obtained by coping with these problems. Performance characterization and composition design are critical to the engineering application of alkali-activated materials. However, it is challenging to characterize reasonable performance and determine the optimal composition due to the complex mechanism and the complex relationship between the performance and the corresponding composition.

In order to characterize the performance of alkali-activated materials, researchers have developed various empirical models based on statistical and regression technology. Cong et al. established a constitutive model to predict the engineering performance of alkali-activated GGBFS/FA concrete [8]. Le et al. used the modified versions of Feret’s and De Larrard’s models to predict the compressive strength of geopolymer recycled aggregate concrete [9]. Zhang et al. propose two empirical equations to predict the residual compressive strength of geopolymer concrete at different temperatures. The error between the prediction results of the proposed equation and the experimental results is very small [10]. Thomas and Peethamparan proposed a formula that uses the compressive strength of alkali-activated concretes to predict their tensile strength and modulus of elasticity [11]. These empirical models can be used to predict engineering performance, such as the strength of alkali-activated materials, and to characterize the relationship between the composition and performance of alkali-activated materials. However, establishing the model requires much experimental support, so it cannot be widely used. Meanwhile, these models are not easy to adjust and optimize according to the actual situation during use, resulting in inaccurate prediction results. Therefore, there is a need for a performance characterization method that does not rely on experiments and can better predict the performance of materials.

With the development of artificial intelligence, machine learning algorithms are often used to predict the performance of various materials [12,13]. Ahmad et al. used Gene Expression Programming (GER) and Artificial Neural Networks (ANNs) to predict the compressive strength of concrete containing recycled coarse aggregates. The results show that the GER model predicts more accurately than the ANN model [14]. Song et al. researched the compressive strength of ceramic waste concrete based on the prediction by the ANN method, and the ANN model showed satisfactory performance for the prediction [15]. Li et al. supposed a stacked ensemble learning model through the eXtreme Gradient Boosting (XGBoost) model of the primary learner, which successfully integrated the prediction output of the primary learning machine, improved the prediction accuracy of the model, and accurately predicted the compressive strength of rice husk ash concrete [16]. Mansouri et al. used CatBoost, Extra-Trees Regressors (ETRs), and Gradient Boosting Regressors (GBRs) to form a hybrid model to estimate the compressive strength of geopolymer concrete more accurately [17]. However, due to the “black box” nature, it is difficult to understand the working mechanism behind the algorithm, and it is impossible to estimate each feature’s importance to predict the output of the model [18]. Therefore, explainable machine learning algorithms are more helpful in understanding the working mechanism between complex relationships [19,20]. As an open-source gradient boosting library, CatBoost consistently has high accuracy and computational speed in various datasets. In addition, it can also be interpreted in combination with PDPs [21,22] and SHAPs [23] to evaluate the contribution of input variables to the prediction results and the marginal effect of one or two variables on the prediction. Therefore, using CatBoost as an alternative model to characterize the complex relationship between the composition and performance of alkali-activated materials is reasonable. However, it is difficult to determine the composition when preparing alkali-activated materials, as their performance depends on complex factors. Material design is critical to the performance of an engineered structure, which depends on the material’s composition and performance [24]. In order to achieve the desired properties, the material’s composition needs to be designed properly. Steinerova optimized the engineering performance of metakaolinite alkali-activated mortar by changing the ratio of alkali-activated binder to filler [25]. Provis et al. and Deventer et al. predicted the use of particle technology to optimize and design the performance of alkali-activated binders and concrete [26,27]. Bagheri et al. optimized the composition of fly ash-based geopolymers with reactive granulated blast furnace slag aggregates by using the Taguchi method [28]. The production of alkali-activated mortar and concrete is also primarily based on the tentative trial-and-error method of Fernández-Jiménez et al. [29]. Although many studies have been conducted on alkali-activated materials, there is still a lack of reasonable composition design methods due to the complexity of the influencing factors of material performance, which hinders the application of alkali-activated materials in engineering.

Recently, optimization techniques based on machine learning algorithms have been gradually applied to determine the optimal composition of materials, which is a potential research method for predicting the performance and composition design of materials [30,31,32,33,34]. Huang et al. established a multi-objective optimization model combining a tree ensemble learning algorithm and a nondominant ordering genetic algorithm (NSGA-II), which improved the efficiency of geopolymer design [35]. Wang et al. proposed an intelligent mixed design method for recycled brick aggregate concrete (RBAC), which integrated many machine learning models to predict the compressive strength of RBAC and used a multi-objective optimization model to obtain the optimal design scheme of the RBAC mixing ratio [36]. Based on machine learning and Particle Swarm Optimization (PSO) algorithms, Li et al. developed an optimal design model of alkali-activated slag–fly ash geopolymer concrete considering 28 days of compressive strength, cost, and carbon emissions [37]. The intelligent optimization algorithm based on machine learning provides a valuable tool for the composition design of materials. However, due to the complexity of the alkali-activated material process, these intelligent optimization methods also have some limitations, such as overfitting, missing the global optimum, and non-convergence. The CatBoost model was used to characterize the relationship between material and performance in this study; it has the advantages of excellent performance, better robustness, and ease of use. The SHGO algorithm is a general-purpose global optimization algorithm based on applications of simplicial integral homology and combinatorial topology. It has excellent convergence and performance on nonlinear objective function and constraint problems. Therefore, it is reasonable to speculate that the optimal composition of the alkali-activated materials can be found based on the CatBoost model and SHGO.

The purpose of this study was to characterize the complex relationship between the composition and performance of slag desulfurization gypsum-based alkali-activated materials so as to determine the optimal composition of the material. The significant difficulties and challenges of this work are analyzing the effect of the material’s composition on its performance and determining the optimal composition to meet multiple target performances of the material using optimization techniques. This study developed a novel framework for performance characterization and composition design by combining CatBoost, SHGO, and laboratory tests to determine the optimal composition of slag desulfurization gypsum-based alkali-activated materials. A laboratory test was used to generate the test data for the different compositions. Based on the test data, CatBoost was employed to capture the complex nonlinear relationship between material compositions and corresponding performance. SHGO was leveraged to search for the optimal composition based on the CatBoost model. The rest of this study is organized as follows. Firstly, the preparation process of slag desulfurization gypsum-based alkali-activated materials and the processing of the experimental results are introduced in Section 2. CatBoost and SHGO are briefly introduced, and the implementation process of the developed framework is described in Section 3. The developed performance characterization and compositional design model based on CatBoost and SHGO are applied to the slag-desulfurized gypsum-based alkali-activated materials for illustration and verification in Section 4. Finally, a summary and the conclusions are listed in Section 5.

## 2. Materials and Methods

Sodium silicate (Na_2_SiO_3_), as an alkaline activator, can give the material a high compressive strength, but the shrinkage of the material is also high [38,39]. Using Ca(OH)_2_ in alkali-activated materials can affect the pore structure and hydration products, change the early development of its mechanical performance, and simultaneously reduce the material’s drying shrinkage [40]. In this study, Na_2_SiO_3_ was used as the main alkaline activator to prepare slag–desulfurization gypsum-based alkali-activated materials, NaOH was used to adjust the modulus of Na_2_SiO_3_, and Ca(OH)_2_ was added to improve the early strength of the materials and reduce the dry shrinkage rate of the materials. Slag, fly ash, and desulfurization gypsum were used as precursors to prepare alkali-activated materials. Twenty-five groups of experiments were designed using orthogonal experimental methods. In order to study the early strength of the slag–desulfurization gypsum-based alkali-activated material, the flexural strength (1 day, 3 days, and 7 days) and compressive strength (1 day, 3 days, and 7 days) of the alkali-activated materials were measured according to the corresponding code and standard.

### 2.1. Test Materials

**Figure 1:**
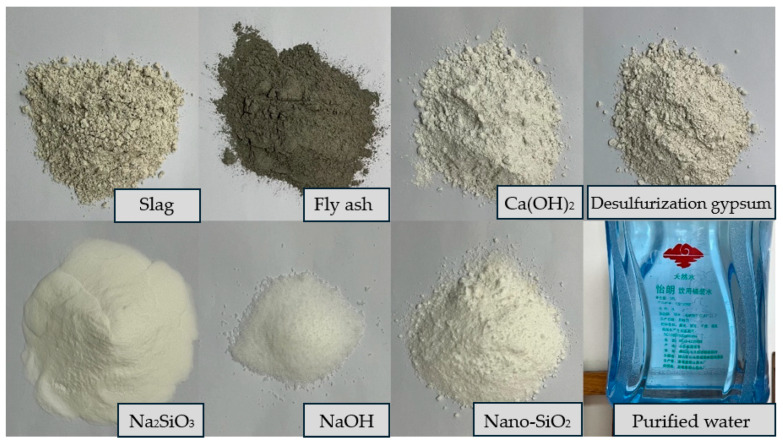
Materials.

The raw materials involved in this study are as follows: slag, fly ash, Ca(OH)_2_, desulfurization gypsum, Na_2_SiO_3_, NaOH, nano-SiO_2_, and water, as shown in Figure 1. The purpose of incorporating nano-SiO_2_ is to improve the strength of the material. In this study, the S95 slag came from Gongyi Longze Water Purification Materials Co., Ltd., Gongyi City, China, which meets the requirements of the standard “Ground granulated blast furnace slag used for cement, mortar and concrete” (GB/T18046-2017) [41]. The performance indices of fly ash used in this test meet the requirements of the “Fly ash used for cement and concrete” (GBT1596-2017) standard [42], and it came from Boheng Mineral Products Trading Co., Ltd., Lingshou, China. The Ca(OH)_2_ is selected from Jinan Xin Kaiming Chemical Co., Ltd., Jinan, China. Its physical and mechanical properties are listed in Table 1 and its chemical composition is listed in Table 2. Desulfurization gypsum and nano-SiO_2_ in this study came from Shandong Guangyuan Chemical Co., Ltd., Dezhou, China and Shanghai Yiyi Alloy Materials Co., Ltd., Shanghai, China, respectively. Their technical characteristics are listed in Table 3. Na_2_SiO_3_ is produced by Henan Borun Foundry Materials Co., Ltd., Qugou, China, the content of SiO_2_ and Na_2_O is 53.52% and 26.75%, respectively, and the modulus is 2. The analytical pure NaOH produced in Tianjin Aopusheng Chemical Co., Ltd., Tianjin, China, is used to adjust its modulus. The water used in the laboratory is purified, which came from Zibo Huilang Mountain Spring Water Plant, Zibo, China

### 2.2. Test Design

In order to study the flexural strength and compressive strength of slag–desulfurization gypsum-based alkali-activated materials, six factors were selected as the main influencing factors, including desulfurization gypsum, Ca(OH)_2_, fly ash, the water–glue ratio, and the content and modulus of the alkaline activator. This study used the orthogonal experimental method to determine the test scheme. The orthogonal experimental factors and their levels are listed in Table 4. In the test, the sum of the mass of slag, Ca(OH)_2_, fly ash, desulfurization gypsum, and nano-SiO_2_ is quantitative. The test level of Ca(OH)_2_ is the ratio of its mass to this quantitative. The remaining part is the total amount of slag, while nano-SiO_2_, fly ash, and desulfurization gypsum can be regarded as the substitutes of total slag, the level of which is the percentage of its mass in the total amount of slag, and the actual amount of slag used is the total amount of slag minus the mass of the above substitutes. It is necessary to mention that the mass of nano-SiO_2_ added to each group of experiments is 1% of the mass of the total slag. In addition, the alkali content is the content of Na_2_O, which is additionally added, and the level is the ratio of its mass to the above quantification. Twenty-five test schemes with different compositions were obtained using the orthogonal design table of six factors and five levels to ensure that there were sufficient test data to analyze the complex relationship between the material’s composition and its performance. The orthogonal experimental design is shown in Table 5. 

The mass of NaOH and Na_2_SiO_3_ can be obtained as follows:

(1) $$\begin{array}{c} m=m_{r}\times a\\ m_{1}=\frac{m\times M}{C_{1}}\\ \frac{2\times 40}{62}=\frac{m_{1}\times C_{2}}{m_{2}} \end{array}$$

where $m_{r}$ is the mass of the sum of the mass of slag, Ca(OH)_2_, fly ash, desulfurization gypsum, and nano-SiO_2_, m and a are the dosage and content of Na_2_O, M is the required modulus, $m_{1}$ and $m_{2}$ are the masses of Na_2_SiO_3_ and NaOH, respectively, and $C_{1}$ and $C_{2}$ are the contents of SiO_2_ and Na_2_O in the Na_2_SiO_3_ used. The specific dosage of the experiment is shown in Table 6.

### 2.3. Specimen Preparation and Performance Measurement

**Figure 2:**
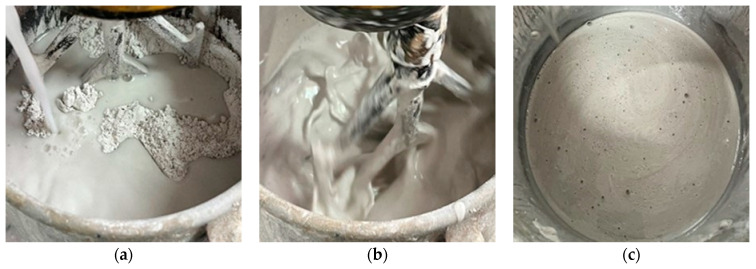
The preparation process of slag–desulfurized gypsum-based alkali-activated materials: (**a**) the mixed materials; (**b**) stirring of the mixture; (**c**) the alkali-activated materials.

In the test, the mass of Na_2_SiO_3_ and NaOH was calculated according to the added amount and modulus of the required alkaline activator. NaOH was added to Na_2_SiO_3_, followed by purified water to prepare an alkaline activator. According to the experimental schemes, various materials were weighed, and then, the alkaline activator and mixed materials were evenly stirred to obtain the alkali-activated materials (Figure 2).

**Figure 3:**
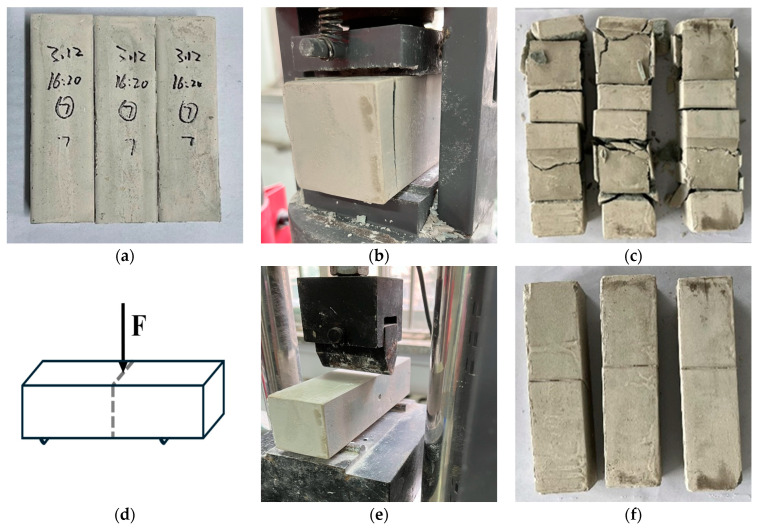
Determination of flexural strength and compressive strength: (**a**) samples; (**b**) compressive strength test; (**c**) specimens after compressive strength test; (**d**) sketch of flexural strength measurements; (**e**) flexural strength test; (**f**) specimens after flexural strength test.

The mixed materials were poured into a 40 mm × 40 mm × 160 mm mold and were cured at 20 ± 2 °C for 24 h to release the mold. The flexural and compressive strengths of the 1-day specimens were determined. The specimens at the age of 3 days and 7 days were placed in a curing room at a temperature of 20 ± 1 °C and a relative humidity greater than 90% until the test age. Then, the automatic bending and compression all-in-one machine was used to measure the flexural strength and the compressive strength of the specimen at the corresponding age according to the specification (Figure 3).

### 2.4. Test Results

The raw data obtained from the experiment often contain a large number of interference factors and cannot truthfully reflect the actual situation if not properly processed due to the complexity of the actual situation, the lack of precision of the observation tools, and the inevitable errors of the observers during testing. Therefore, in order to make the performance of the experimental data more effective and obtain more accurate scientific conclusions, it is necessary to analyze and process these raw data. According to the “Testing Methods of Cement and Concrete for Highway Engineering” (JTG3420-2020) [43], the test results of the flexural strength and compressive strength of slag–desulfurization gypsum-based alkali-activated materials were processed, and the final results are listed in Table 7.

**Figure 4:**
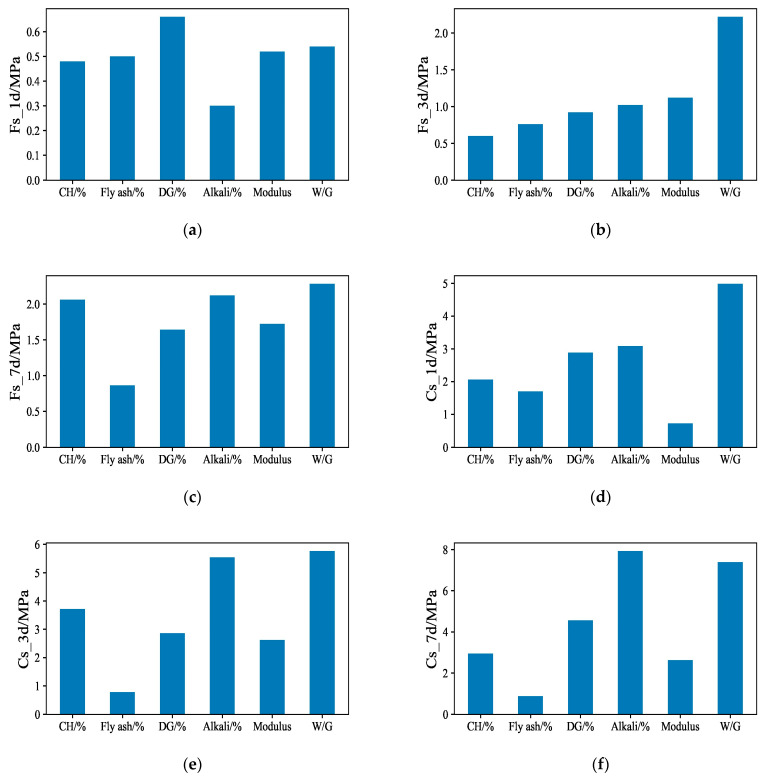
The range of each performance: (**a**) FS_1d; (**b**) FS_3d; (**c**) FS_7d; (**d**) CS_1d; (**e**) CS_3d; (**f**) CS_7d.

**Figure 5:**
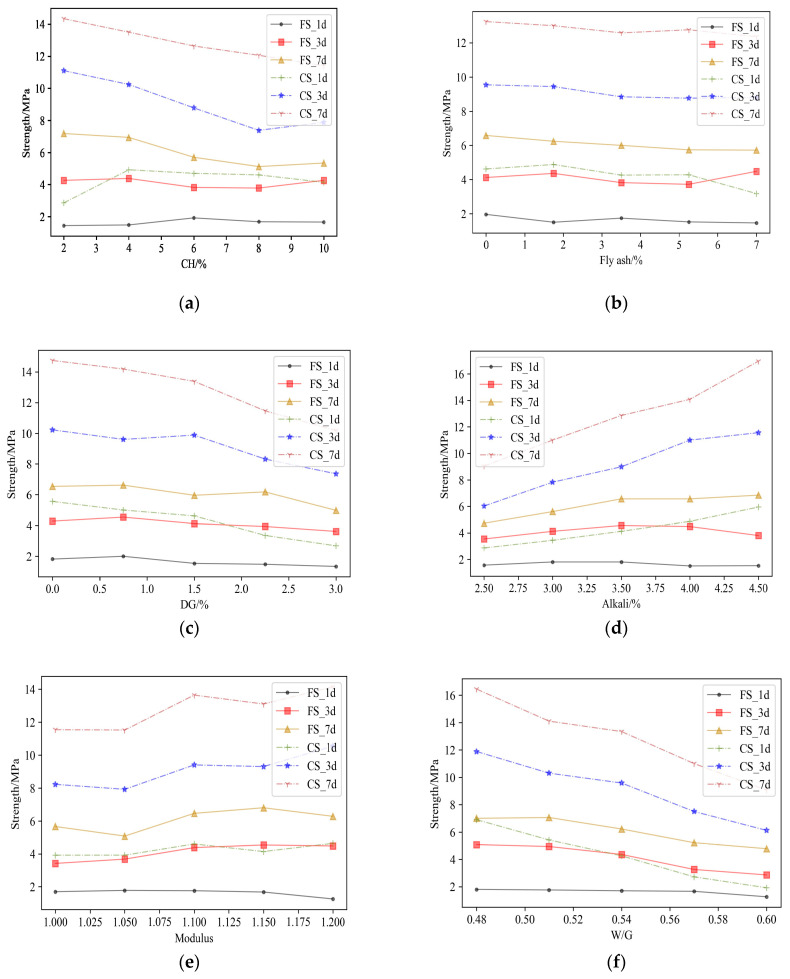
The influence of each factor on the strength of the material: (**a**) Ca(OH)_2_; (**b**) fly ash; (**c**) DG; (**d**) alkali; (**e**) modulus; (**f**) W/G.

Range analysis is the most commonly used method for the analysis of orthogonal experimental results. According to Table 7, the ranges for each experimental factor and the corresponding average values for each experimental level are calculated to analyze the orthogonal experimental results. Figure 4 shows the range of the materials’ performance and the main and secondary factors affecting the flexural and compressive strength at different ages. Figure 5 shows the trend in the influence of each experimental factor on the flexural and compressive strength of the material at different ages. The optimal composition can be obtained according to orthogonal experiments by combining Figure 4 and Figure 5 and is listed in Table 8.

## 3. Performance Characterization and Composition Design Methods

In order to determine the relationship between the composition and performance of slag–desulfurization gypsum-based alkali-activated materials, an effective method needs to be considered. However, the relationship between the performance and the composition of alkali-activated materials is complex, and performance characterization is also very difficult. This study used the CatBoost model to characterize the complex relationship between the compositions and performance of slag–desulfurized gypsum-based alkali-activated materials. In order to determine the optimal material composition, SHGO was used to search for the optimal composition of the slag–desulfurized gypsum-based alkali-activated materials based on the CatBoost performance model.

### 3.1. CatBoost

**Figure 6:**
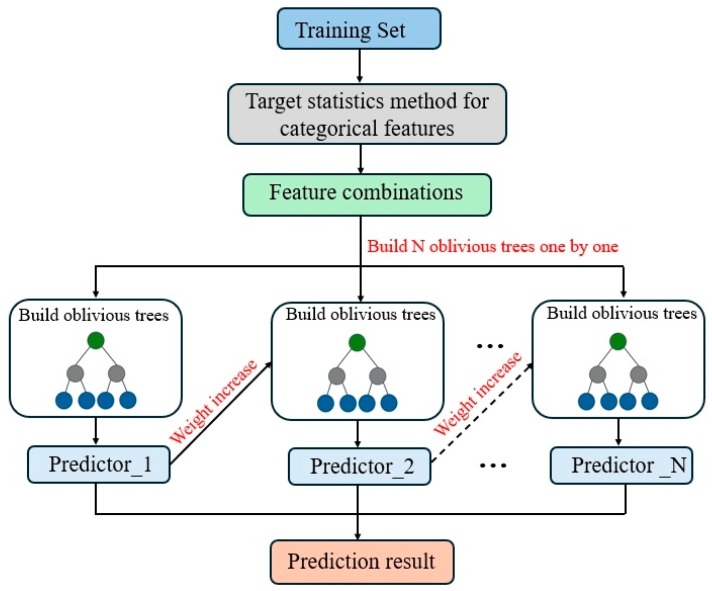
Structure of CatBoost.

CatBoost is a new type of gradient boosting technology (GBDT) proposed by Yandex, which is different from other gradient boosting algorithms and is suitable for small datasets and many types of data [44]. CatBoost uses an oblivious binary tree characterized by the fact that each layer is split using the same features (Figure 6) [45]. It has a specific regular effect on the constraints on the tree structure. More importantly, it allows for extremely fast inference of CatBoost models. During the CatBoost tree prediction process, the splits of each feature are disordered and independent, allowing for multiple ones to be predicted at the same time.

CatBoost was designed to better handle categorical features in GBDT features. The method is known as Greedy Target-based Statistics or Greedy TS. Let us assume that $D=\{{\left(X_{i},Y_{i}\right\}}_{i}=1,\ldots ,n$, where $X_{i}=(x_{i,1},\ldots ,x_{i,m})$, $Y_{i}\in R$ is a label value, and m is features that are mixed, some numeric and some categorical.

(2) $${\hat{x}}_{k}^{i}=\frac{\sum_{j=1}^{n}[x_{j,k}=x_{i,k}]\cdot Y_{i}}{\sum_{j=1}^{n}[x_{j,k}=x_{i,k}]}$$

However, the usual features of this method contain more information than the tags used for substitution. In this case, if the feature is represented by the average value of the labels, the problem of conditional drift may occur.

By adding a prior distribution term, the influence of noise and low-frequency data on the data distribution can be reduced, thereby improving Greedy TS:

(3) $${\hat{x}}_{k}^{i}=\frac{\sum_{j=1}^{p-1}[x_{\sigma_{j},k}=x_{\sigma_{p},k}]Y_{\sigma_{j}}+a\cdot P}{\sum_{j=1}^{p-1}[x_{\sigma_{j},k}=x_{\sigma_{p},k}]+a}$$

CatBoost uses a novel method to calculate the value of leaf nodes which can avoid the problem of overfitting in direct calculations in the arrangement of multiple datasets and then improve the accuracy and generalization ability of the algorithm [46].

### 3.2. SHGO

The simplicial homology global optimization (SHGO) algorithm is a general-purpose global optimization algorithm based on applications of simplicial integral homology and combinatorial topology. SHGO approximates the homology groups of a complex built on a hypersurface homeomorphic to a complex on the objective function. This provides both approximations of locally convex subdomains in the search space through Sperner’s lemma and a useful visual tool for characterizing and efficiently solving higher-dimensional black- and gray-box optimization problems [47]. In this study, SHGO was used to solve the optimization problem of slag–desulfurization gypsum-based alkali-activated materials’ composition.

### 3.3. Performance Characterization and Composition Design Based on CatBoost and SHGO

**Figure 7:**
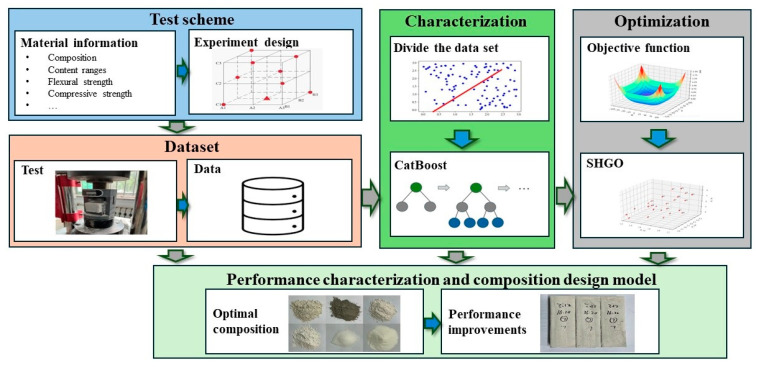
Flowchart of developed performance characterization and composition design framework.

This study developed a novel framework for the performance characterization and composition design of slag–desulfurization gypsum-based alkali-activated materials by combining CatBoost, SHGO, and laboratory tests. A laboratory test was used to generate the composition data and the corresponding performance. CatBoost was adopted to build a complex relationship between composition and performance and characterize the material based on the test data. SHGO was used to determine the optimal composition based on the CatBoost performance characterization model. The flowchart of the framework is shown in Figure 7.

Step 1: Collect the relevant information on slag–desulfurization gypsum-based alkali-activated materials and determine their main compositions and key performance based on the engineering requirement.
Step 2: Determine the test scheme based on the experimental design method.
Step 3: Implement the test scheme and obtain the materials’ performance.
Step 4: Analyze the test results and generate the samples for the CatBoost algorithm.
Step 5: Characterize the performance based on the CatBoost model and test data.
Step 6: Generate the optimization model based on the CatBoost-based performance characterization model.
Step 7: Build the composition design model based on the CatBoost-based performance characterization and SHGO.
Step 8: Obtain the optimal composition based on the CatBoost, SHGO, and tests.

## 4. Application

In order to illustrate and validate the developed framework, the performance and composition of the slag–desulfurization gypsum-based alkali-activated materials were evaluated and determined. The performance and composition of each factor were investigated based on the CatBoost-based performance characterization model. The optimal composition was also compared with the traditional method. The CatBoost model characterizes the performance well and reveals the complex relationship between the performance and the corresponding composition of the slag–desulfurization gypsum-based alkali-activated materials. The developed framework provides an excellent way to characterize material performance and determine the optimal composition.

### 4.1. Performance Characterization

**Figure 8:**
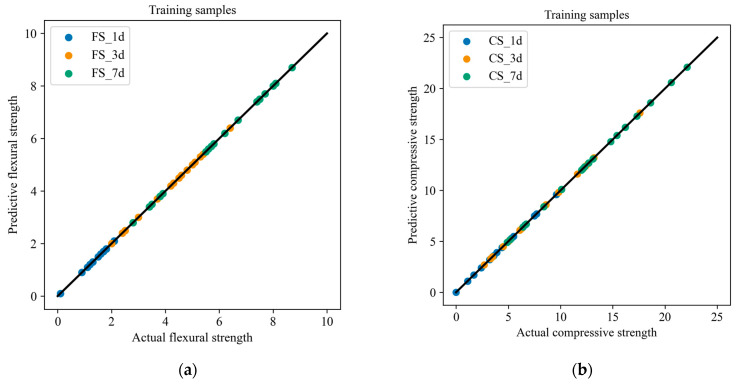
Comparisons between the experimental and predicted results of the training samples: (**a**) flexural strength; (**b**) compressive strength.

**Figure 9:**
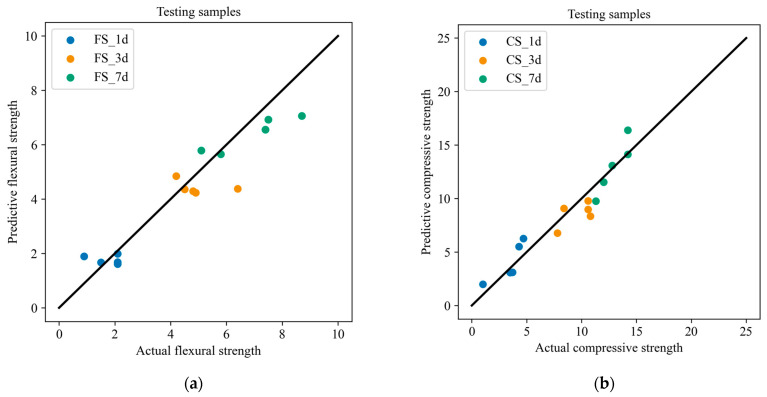
Comparisons between the experimental and predicted results of the testing samples: (**a**) flexural strength; (**b**) compressive strength.

**Figure 10:**
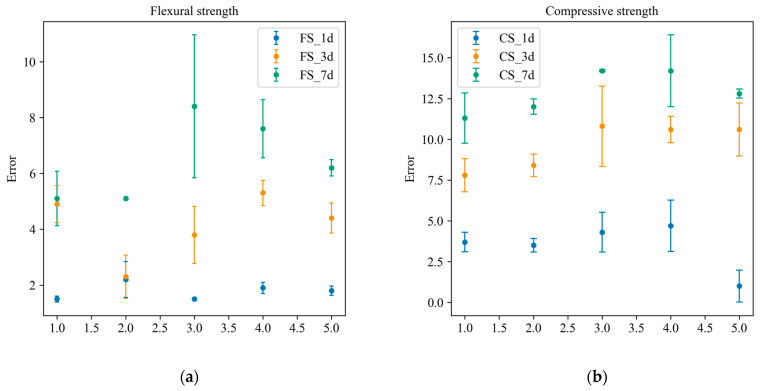
The error bar of the experimental and predicted results of the testing samples: (**a**) flexural strength; (**b**) compressive strength.

**Figure 11:**
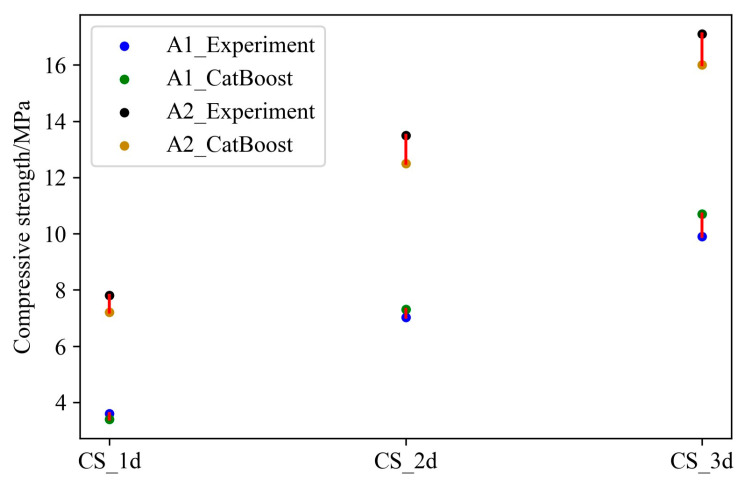
Comparisons between the experimental and predicted results of A1 and A2.

In order to better explore the relationship between the composition and performance of slag–desulfurization gypsum-based alkali-activated materials, the measured twenty-five groups of experimental data were divided into training samples and test samples, and the CatBoost model was used to characterize the performance. Figure 8 compares the actual and predicted results of the training samples. The result shows that the predicted material performance is consistent with the experimental result. In order to illustrate the generalization performance of the developed performance characterization model, the test samples were used to predict the materials’ performance (Figure 9). It can be seen that the flexural and compressive strengths at different ages of the materials predicted by the developed model are in good agreement with the experimental performance. Figure 10 presents the error of the experimental and predicted results of the testing samples. Due to the different range of results for flexural strength and compressive strength at different ages, the results presented in the figure are also different. However, it can be seen that the error is within an acceptable range. In addition, Table 9 lists the compositions of the alkali-activated materials in other combinations (outside of this study), denoted as A1 and A2. Table 10 lists the corresponding performance of these two compositions based on laboratory tests and CatBoost. It can be seen that the relative error of both the predicted and the actual value is less than 9%. Figure 11 shows the comparison of the predicted and actual values. It further demonstrates the excellent predictive ability of CatBoost to accurately predict material performance based on material composition, replacing uneconomical laboratory experiments and providing a scientific and practical way to characterize material performance.

### 4.2. Composition Design

From the above, it can be seen that the compositions of slag–desulfurization gypsum-based alkali-activated materials are complex. Determining the composition is also tricky because their performance depends on complex factors. For slag–desulfurization gypsum-based alkali-activated materials, the relationship between the materials’ compositions and performance is more complex and highly nonlinear due to the increase in the materials’ compositions, and an effective method needs to be considered in order to determine the relationship between the materials’ compositions and alkali-activated materials’ performance. This study used SHGO to search for the optimal composition based on the CatBoost performance characterization model. 

Taking the maximum flexural strength and compressive strength of slag–desulfurization gypsum-based alkali-activated materials at different ages as the goal, an optimization problem was proposed, and the minimum value of the objective function was found by using SHGO. The optimization problems supposed in this study are as follows:

(4) $$\begin{array}{l} \underset{x}{\min}f(x)=\frac{1}{\sigma_{1}\left(X\right)+\sigma_{3}\left(X\right)+\sigma_{7}\left(X\right)+\sigma_{1}^{\prime }\left(X\right)+\sigma_{3}^{\prime }\left(X\right)+\sigma_{7}^{\prime }\left(X\right)},x\in R^{n}\\ \begin{array}{cccc} s.t. & & \sigma_{1}\left(X\right)\leq \sigma_{3}\left(X\right) & \\ & & \sigma_{3}\left(X\right)\leq \sigma_{7}\left(X\right) & \\ & & \begin{array}{l} \sigma_{1}^{\prime }\left(X\right)\leq \sigma_{3}^{\prime }\left(X\right)\\ \sigma_{3}^{\prime }\left(X\right)\leq \sigma_{7}^{\prime }\left(X\right)\\ 2.00<x_{1}<10.00 \end{array} & \\ & & 0.00<x_{2}<7.00 & \\ & & 0.00<x_{3}<0.30 & \\ & & 2.50<x_{4}<4.50 & \\ & & 1.00<x_{5}<1.20 & \\ & & 0.48<x_{6}<0.60 & \end{array} \end{array}$$

where f(x) is the objective function and $\sigma_{1}\left(X\right),\sigma_{3}\left(X\right),\sigma_{7}\left(X\right),\sigma_{1}^{\prime }\left(X\right),\sigma_{3}^{\prime }\left(X\right)$, and $\sigma_{7}^{\prime }\left(X\right)$ represent the flexural strength and compressive strength of the slag–desulfurization gypsum-based alkali-activated materials at 1 day, 3 days, and 7 days, respectively. $x_{1},x_{2},x_{3},x_{4},x_{5},x_{6}$ represent theCH, fly ash, DG, and alkali content, the modulus, and the W/G, respectively. The flexural strength and compressive strength $\sigma_{1}\left(X\right),\sigma_{3}\left(X\right),\sigma_{7}\left(X\right),\sigma_{1}^{\prime }\left(X\right),\sigma_{3}^{\prime }\left(X\right)$, and $\sigma_{7}^{\prime }\left(X\right)$ were obtained by using the CatBoost performance characterization model. SHGO was used to search for the optimal value of the objective function and determine the optimal composition.

**Figure 12:**
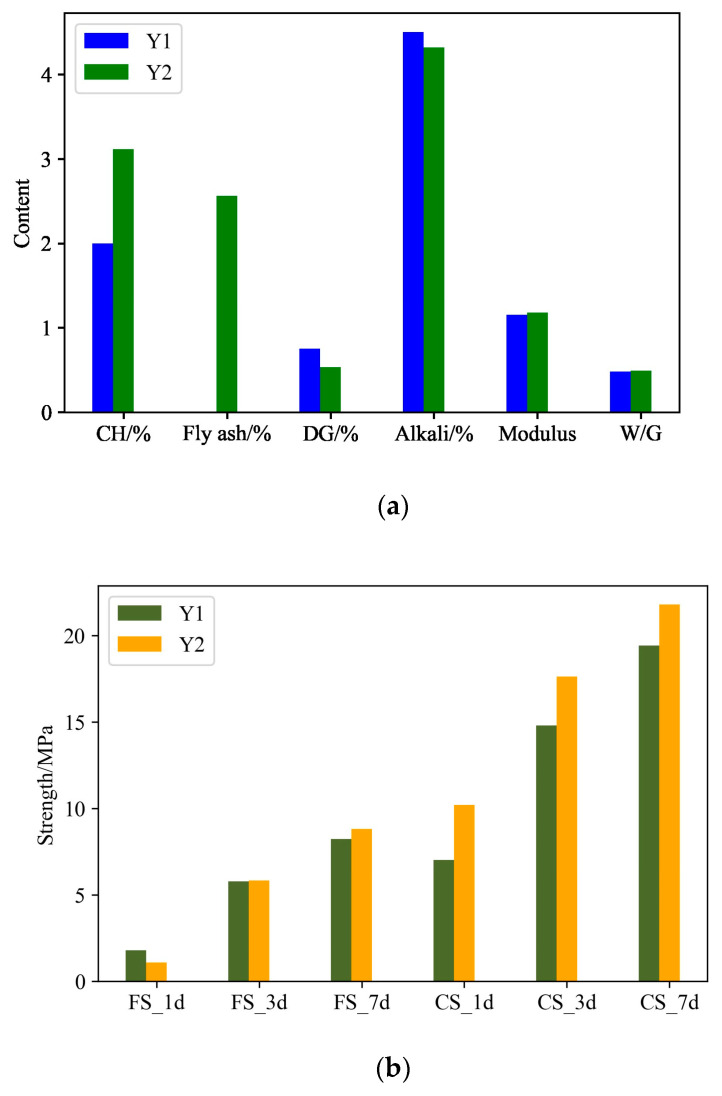

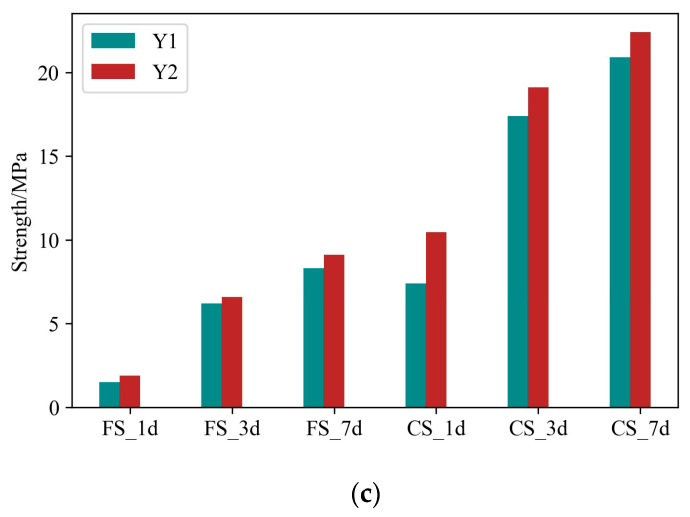
A comparison of optimal composition between SHGO and the orthogonal experiments: (**a**) a comparison of the composition between Y1 and Y2; (**b**) a comparison of the predicted strength by CatBoost between Y1 and Y2; (**c**) a comparison of the experimental strength between Y1 and Y2.

**Figure 13:**
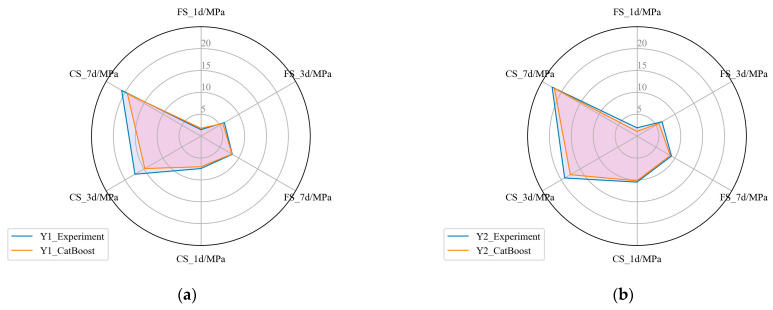
A comparison between the predicted strength by CatBoost and the experimental strength: (**a**) a comparison of Y1 between the predicted strength by CatBoost and the experimental strength; (**b**) a comparison of Y2 between the predicted strength by CatBoost and the experimental strength.

The optimal composition obtained by the traditional orthogonal experiments and proposed method are denoted as Y1 and Y2, respectively. Table 11 lists Y1 and Y2. Table 12 lists the predicted strength based on the CatBoost performance characterization model and the strength measured by the laboratory experiments. As can be seen from Figure 12, Y1 and Y2 are relatively close, and the performance of Y2 is better than Y1, predicted based on the CatBoost model, which was verified by the results of the laboratory tests. Compared with Y1, the actual flexural strength and compressive strength of Y2 at 1, 3, and 7 days increased by 26.67%, 6.45%, 9.64%, 41.89%, 9.77%, and 7.18%, respectively. It can be seen that the developed composition design framework based on the CatBoost model and SHGO optimal technology is feasible and scientific, which provides an effective tool for the composition design of materials. In addition, Figure 13 shows a comparison of the predicted strength by the CatBoost performance characterization model and the experimental strength between Y1 and Y2. And the predicted strength and experimental strength of the two compositions are very consistent. This again illustrates that the CatBoost performance characterization model can well capture the complex mechanism between material composition and performance and can be used as an alternative to laboratory experiments to characterize material performance.

**Figure 14:**
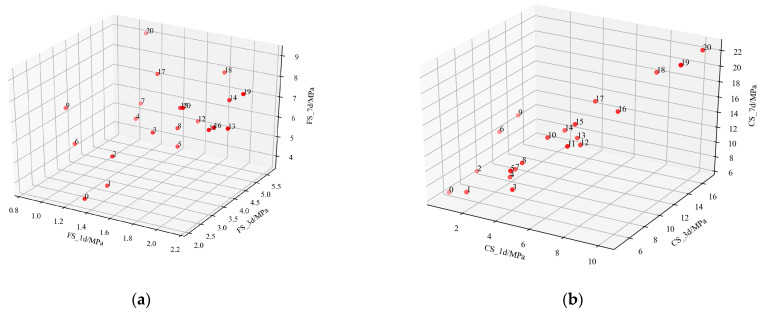
The search process for flexural strength and compressive strength: (**a**) the search process for flexural strength; (**b**) the search process for compressive strength.

**Figure 15:**
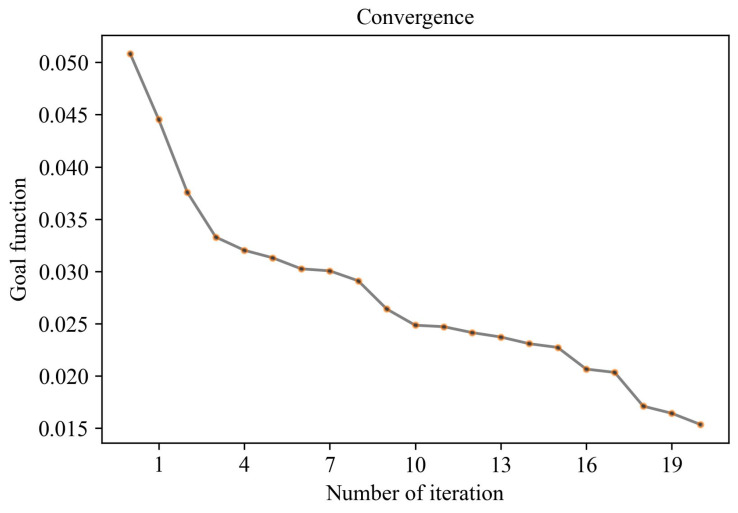
Convergence of objective function.

The SHGO method is used to search for the optimal composition, and the process is visualized below. Figure 14 clearly shows the optimized process of the flexural strength and compressive strength of slag–desulfurization gypsum-based alkali-activated materials at different ages in searching for the optimal composition. It shows that it is difficult to obtain the optimal composition due to the conflict between the various factors. Figure 15 shows the convergence of the objective function of the search process. Each point represents its number of iterations.The proposed method converges well with the increase in the number of iterations and can find the optimal composition of the materials. The above results show that the influence of slag–desulfurization gypsum-based alkali-activated materials on the performance of material composition is very complex, and the CatBoost-based performance characterization model can well characterize this relationship. SHGO is an excellent optimization method that can be used to find the optimal composition of materials based on the CatBoost performance characterization model.

### 4.3. Discussion

In order to investigate the effect of input variables on the performance of slag–desulfurized gypsum-based alkali-activated materials, a correlation plot was used to study the relationship between input variables, and the CatBoost model was explained using SHAPs and PDPs. SHAP evaluates the influence of input factors on material performance. At the same time, a PDP shows the combined effects of one or two input factors on material performance.

#### 4.3.1. Correlation

**Figure 16:**
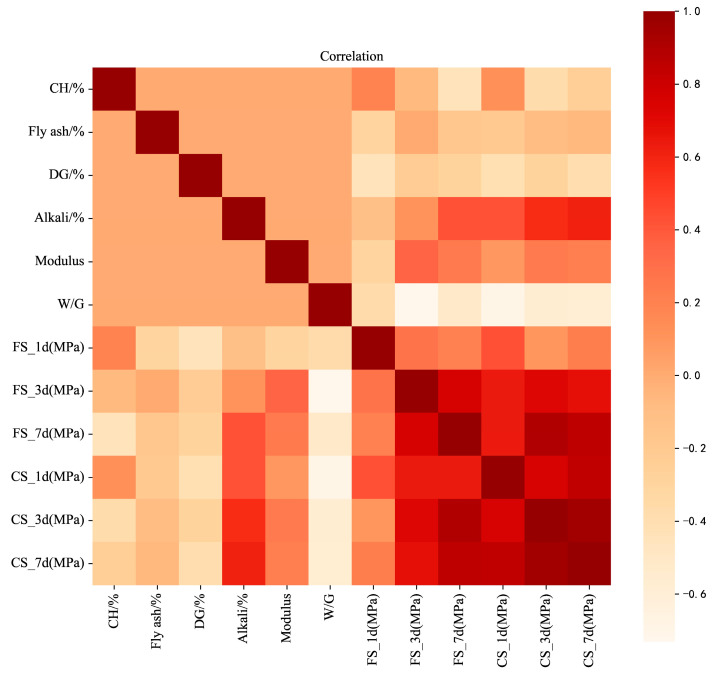
The correlation of the materials’ composition and their performance.

Figure 16 describes the linear relationship between each pair of input and output variables by Pearson correlation coefficients. Orange–red and light pink symbolize positive and negative correlations. It can be seen that the added amount of alkali has a high correlation with the FS_7d, CS_1d, CS_3d, and CS_7d, with coefficients of 0.42, 0.42, 0.57, and 0.61, respectively. There was a negative correlation between the W/G and all of the strengths, and the correlation was strong. The W/G had the most significant effect on the FS_3d, with a coefficient of −0.73. All of the above shows that the composition of slag–desulfurization gypsum-based alkali-activated materials affects its performance. As expected, the correlation between the flexural strength and compressive strength of materials at different ages is obvious and positively correlated, proving the data’s scientific and reliability.

#### 4.3.2. Feature Importance Based on SHAP

**Figure 17:**
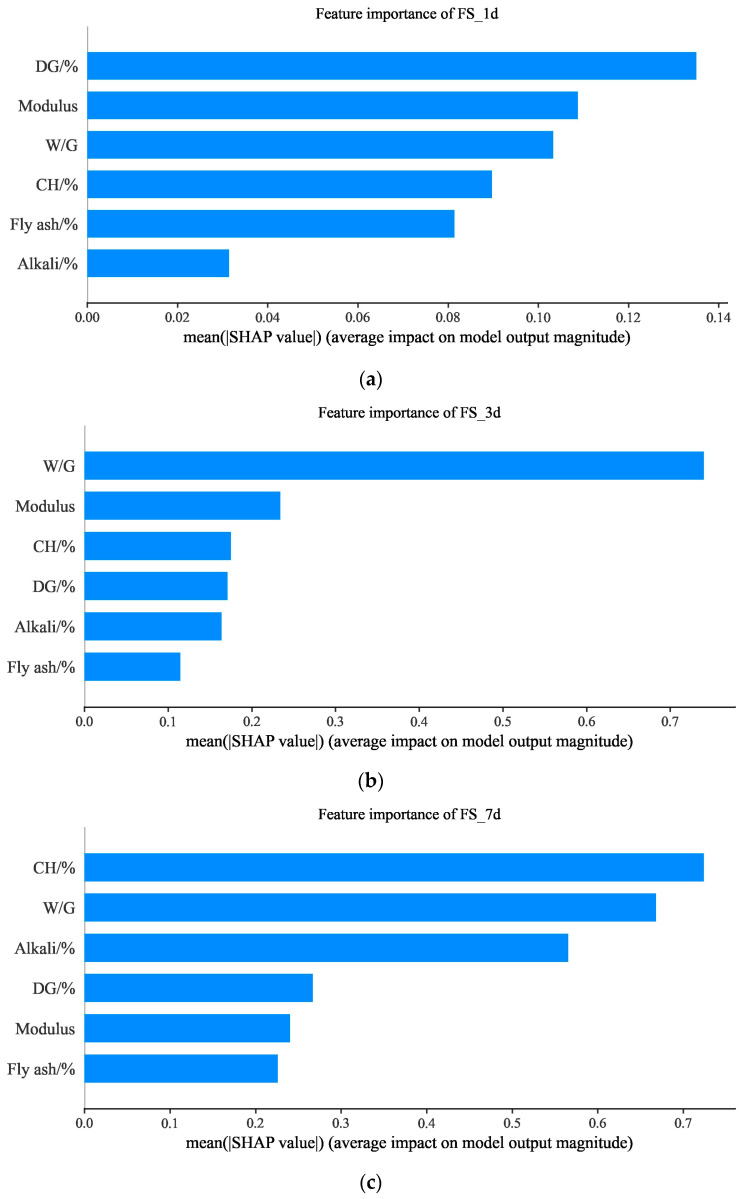

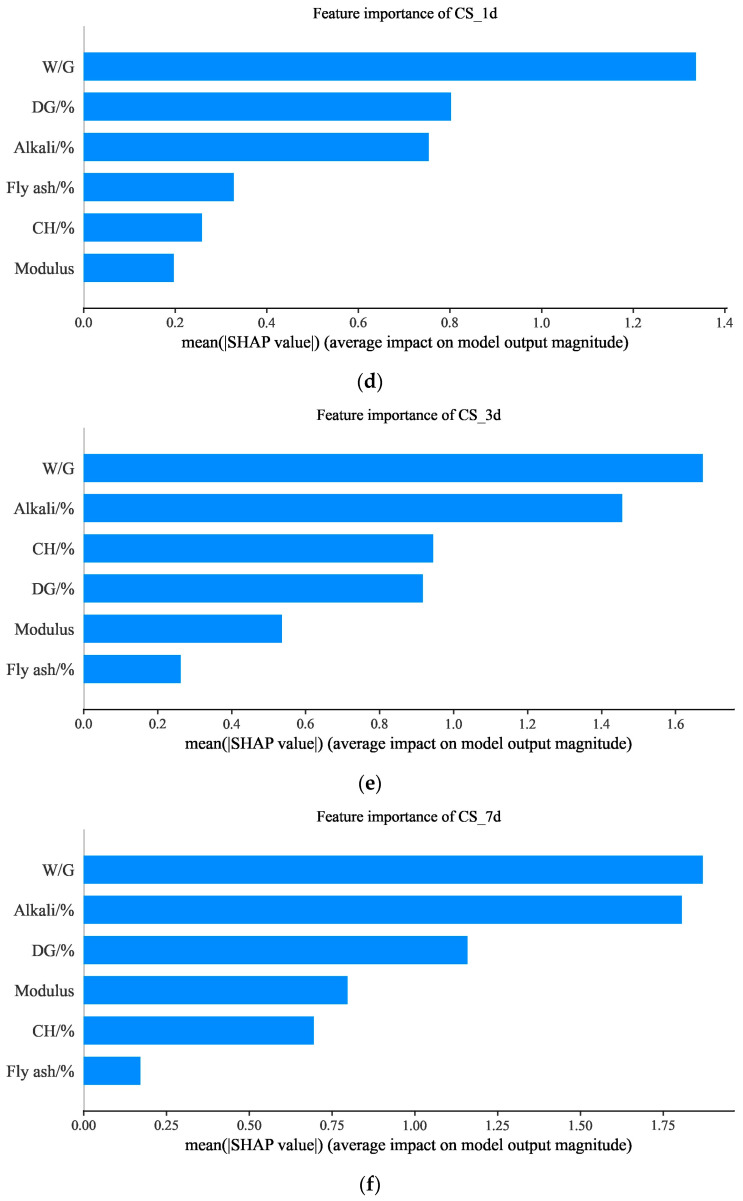
Feature importance of CatBoost-based performance characterization: (**a**) feature importance of FS_1d; (**b**) feature importance of FS_3d; (**c**) feature importance of FS_7d; (**d**) feature importance of CS_1d; (**e**) feature importance of CS_3d; (**f**) feature importance of CS_7d.

In order to elucidate the impact of input variables on model prediction, the method of SHAP analysis is employed in this study [48,49,50]. SHAP can assess feature importance by calculating the mean absolute SHAP value. Figure 17 shows an analysis of the importance of features using SHAP values based on the CatBoost model. As shown in Figure 17, the effects of the input variables on flexural strength and compressive strength at different ages are also different. The most important factors affecting the FS_1d, FS_3d, and FS_7d are the DG content, the W/G, and the CHcontent_._ However, for the compressive strength of slag–desulfurization gypsum-based alkali-activated materials, even with different ages, the most important influencing factor is W/G, which is consistent with the results of Nguyen’s study [51]. And similar to the results of previous studies [7], the alkali content also plays a very important role in it. In addition, it can be seen that fly ash has little effect on both flexural strength and compressive strength.

**Figure 18:**
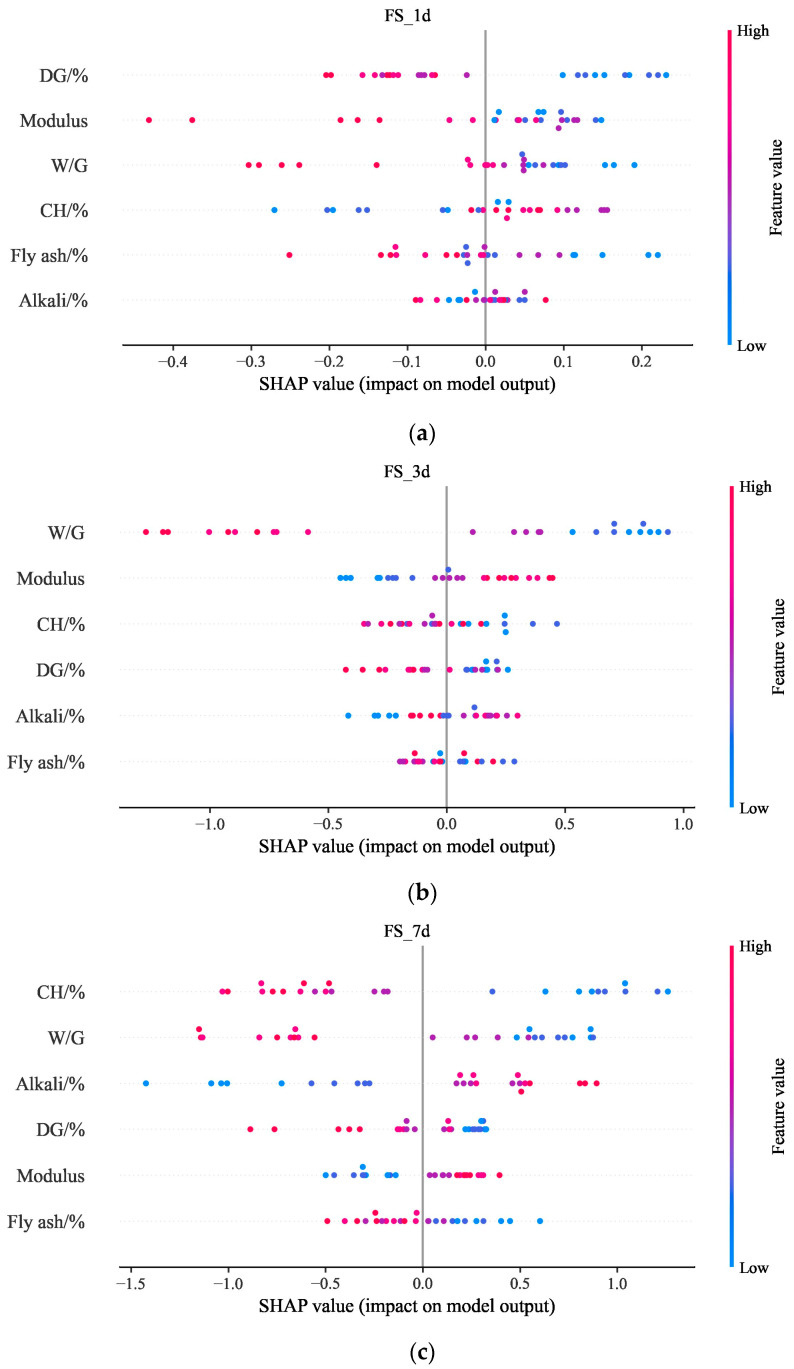

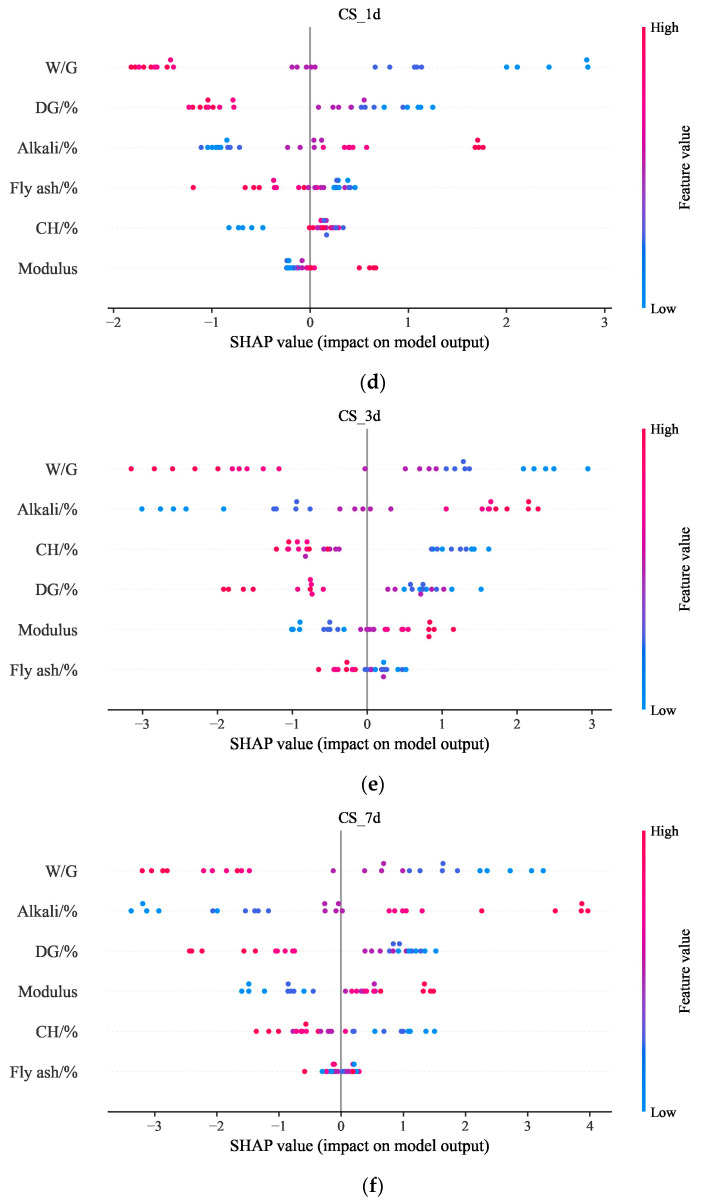
SHAP value summary plot: (**a**) SHAP value summary plot of FS_1d; (**b**) SHAP value summary plot of FS_3d; (**c**) SHAP value summary plot of FS_7d; (**d**) SHAP value summary plot of CS_1d; (**e**) SHAP value summary plot of CS_3d; (**f**) SHAP value summary plot of CS_7d.

Figure 18 shows a summary of the SHAP values for all input variables. Each dot represents the SHAP value for each feature of a particular sample. It should be mentioned that input variables that have a positive effect on the materials’ performance are shown in red and those with a negative effect are in blue. As shown in Figure 18, low DG content and a low modulus tend to increase the FS_1d of slag–desulfurized gypsum-based alkali-activated materials. It can also be seen that a low W/G and low Chcontent tend to increase the FS_3d and FS_7d of the materials. Similarly, a low W/G, low DG content, and a high alkali content lead to improvements in the CS_1d, CS_3d, and CS_7d of slag–desulfurized gypsum-based alkali-activated materials. The high alkali content leads to the formation of silica-rich gels and C-S-H gels which increase the compressive strength of the material [52]. All of the above further illustrate that the relationship between the composition and performance of slag–desulfurization gypsum-based alkali-activated materials is very complex, and the SHAP can explain the mechanism behind it based on the CatBoost model, which characterizes this complex relationship. It also offers a helpful tool to search for the optimal composition of civil materials.

#### 4.3.3. Feature Importance Based on SHAP

**Figure 19:**
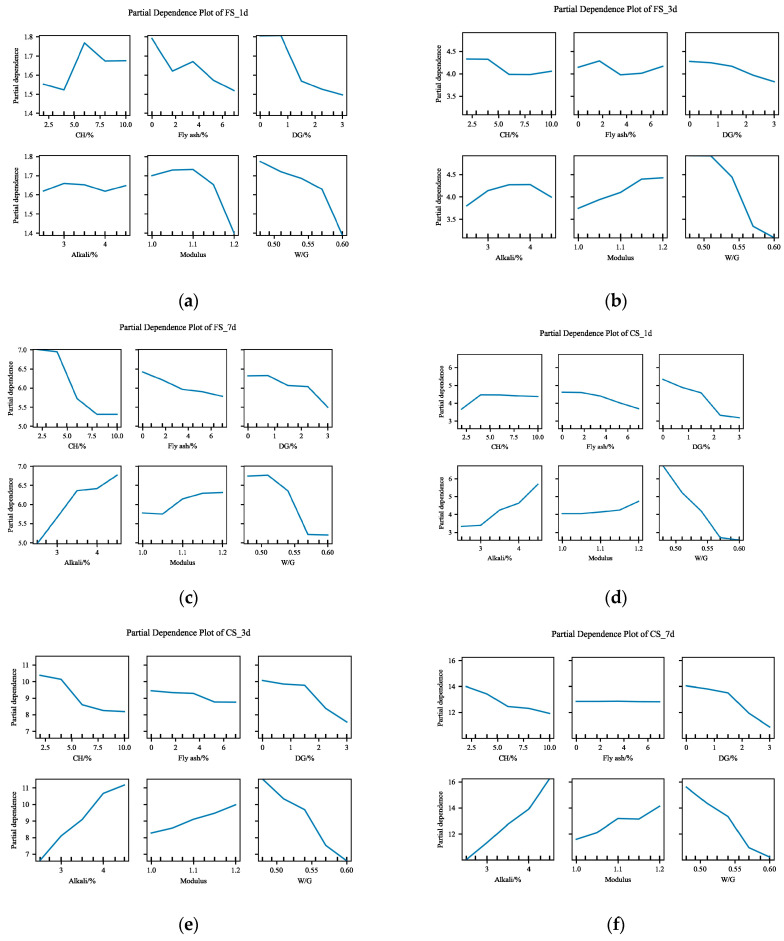
Partial dependence plot: (**a**) partial dependence plot of FS_1d; (**b**) partial dependence plot of FS_3d; (**c**) partial dependence plot of FS_7d; (**d**) partial dependence plot of CS_1d; (**e**) partial dependence plot of CS_3d; (**f**) partial dependence plot of CS_7d.

PDPs can visualize and analyze the interaction between a target and input features. Figure 19 shows the PDPs of the six input variables for the flexural and compressive strength at different ages of the slag–desulfurized gypsum-based alkali-activated materials. From the figure, we can clearly understand the trends in the impact of the six input variables on the materials’ performance. In general, in addition to the FS_1d, the alkali content and modulus have a positive contribution to the other five strengths. Conversely, CHfly ash, and DG content and the W/G have negative effects. Excess CHwill be present in the form of hexagonal plate silicate crystals without gelling, which will reduce the strength [40]. Furthermore, the degree to which different compositions affect the strength can also be obtained from the PDP. For example, the changing trend in fly ash is relatively flat for 1-day, 3-day, and 7-day compressive strength, while W/G shows a strong negative effect on them. The results of the PDP are consistent with the results of the importance of the SHAP feature.

**Figure 20:**
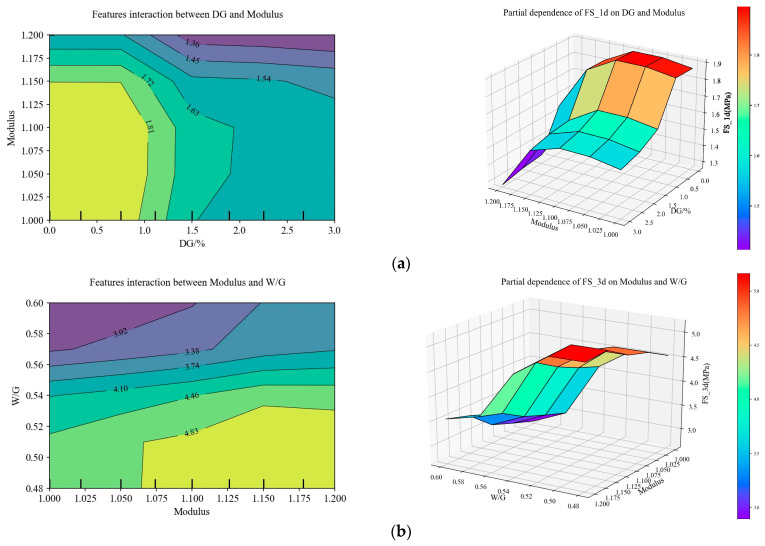

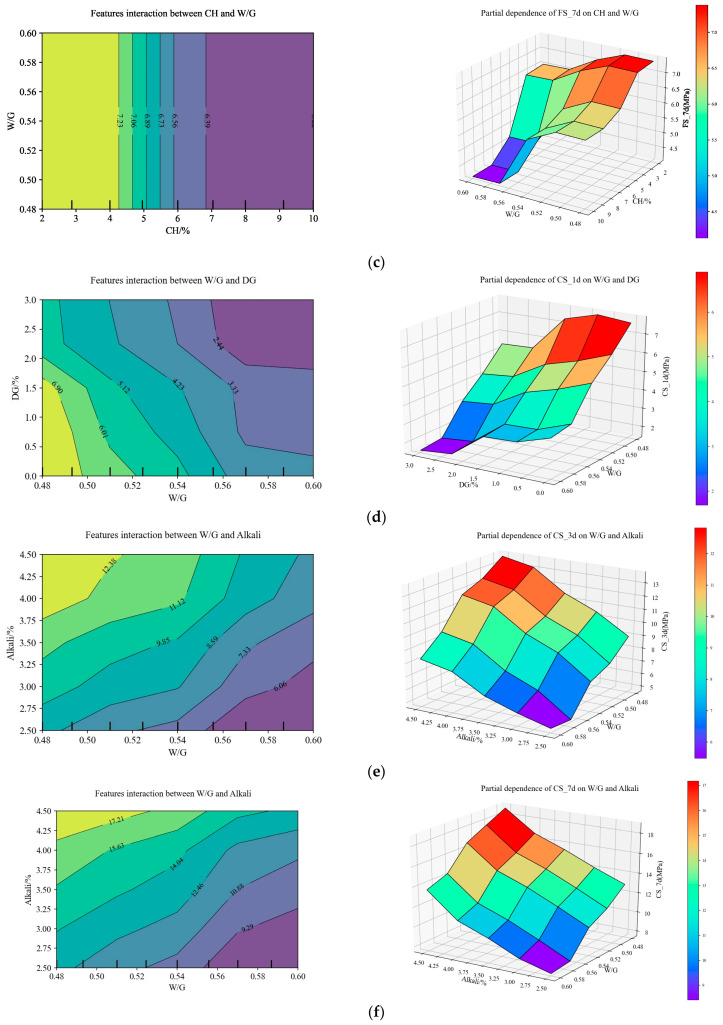
Effects of two main variables on corresponding performance: (**a**) effects of modulus and DG content on FS_1d (2D and 3D); (**b**) effects of modulus and W/G on FS_3d (2D and 3D); (**c**) effects of CHand W/G on FS_7d (2D and 3D); (**d**) effects of DG content and W/G on CS_1d (2D and 3D); (**e**) effects of alkali content and W/G on CS_3d (2D and 3D); (**f**) effects of alkali content and W/G on CS_7d (2D and 3D).

Figure 20 shows the marginal effects of two main factors corresponding to the flexural strength and compressive strength of the slag–desulfurization gypsum-based alkali-activated materials at different ages. The two main factors that affect the corresponding performance can be obtained from Figure 17. The optimal FS_1d can be achieved when the modulus is 1.00–1.15 and the DG content is 0–0.9%. Conversely, when the modulus is 1.18–1.20 and the DG content is 1.4–3%, the FS_1d is extremely low, and the maximum FS_1d value of this space is only 1.36 MPa. Next, it shows the effect of the modulus and W/G on the FS_3d of the materials. When the modulus is 1.06–1.20 and the W/G is 0.48–0.53, the optimal value of FS_3d is 4.83 MPa, while when the modulus is 1.0–1.1 and the W/G is 0.57–0.60, the FS_3d value is the lowest, which is 3.02 MPa. As shown in Figure 20, there is little interaction between the two main influencing factors of FS_7d, CHand the W/G.

For the compressive strength of slag–desulfurization gypsum-based alkali-activated materials, the W/G has a significant negative effect on them. Figure 20 shows the effects of the DG content and the W/G on CS_1d. When the W/G is 0.48–0.50 and the DG content is 0–1.6%, the optimal value of CS_1d can be reached at 6.90 MPa. Conversely, when the W/G is 0.56–0.60 and the DG content is 1.8–3.0%, the value of CS_1d is the lowest, at 2.44 MPa. It can be seen that the effects of the W/G and the alkali content on CS_3d and CS_7d are similar. Specifically, when the optimal value is reached in CS_3d, the alkali content is 3.75–4.5% and the W/G is 0.48–0.515. When the alkali content is 4.15–4.5% and the W/G is 0.48–0.525, the optimal value range of CS_7d is reached. Moreover, when the alkali content is 2.5–3.25% and the W/G is 0.55–0.60, both CS_3d and CS_7d are the lowest, at 6.06 MPa and 9.29 MPa, respectively. The above proves that the relationship between the composition and performance of slag–desulfurization gypsum-based alkali-activated materials is quite complex, and it is challenging to characterize the performance. The CatBoost-based performance characterization model is a substitute model for presenting the relationship. The PDP can explain how the input variables affect the materials’ performance and the effects between the input variables based on the CatBoost model. It provides a solid basis for the composition design of materials.

#### 4.3.4. Potential Applications

The proposed framework for performance characterization and composition design using machine learning and optimal technology works well in optimizing the composition of materials in civil engineering. In civil engineering materials, the material’s performance depends on its composition, and the use of the developed framework can determine the material’s composition to meet the needs of different goals. It is a very fast and effective way to determine the composition for the development of new materials. This improves efficiency and reduces the loss of manpower and financial resources in practical engineering applications, which is of great significance to the development of civil engineering materials. In addition, the interpretable methods were used to analyze the trend and mode of influence of material composition on performance, clarify the direction of material composition optimization, and explain the relationship between composition and performance and between composition and composition, which is helpful to better understand the role of the composition of materials and has guiding significance for the development and research of new civil engineering materials in the future.

## 5. Conclusions

In this study, a novel framework for performance characterization and composition design was developed by combining CatBoost, SHGO, and laboratory tests to find the optimal composition of civil materials. The developed framework is illustrated by slag–desulfurization gypsum-based alkali-activated materials. With the results obtained, it is possible to conclude the following:

- CatBoost is a powerful tool that can characterize the complex relationship between material composition and performance and can predict the performance of slag–desulfurization gypsum-based alkali-activated materials very well, and the predicted strength of the material based on CatBoost is consistent with the results obtained from laboratory tests.
- In this study, SHGO is used to search for the optimal composition of the slag–desulfurization gypsum-based alkali-activated materials to maximize the flexural and compressive strength at different ages. The results show that the optimal composition determined by SHGO is similar to that obtained by traditional experimental methods. The final composition is as follows: Ca(OH)_2_: 3.1%, fly ash: 2.6%, DG: 0.53%, alkali: 4.3%, modulus: 1.18, and W/G: 0.49.
- The performance characterization and composition design framework proposed in this study determines the optimal composition of slag–desulfurization gypsum-based alkali-activated materials that meets the target. Compared with the material composition obtained by traditional experiments, the actual flexural strength and compressive strength at 1, 3, and 7 days increased by 26.67%, 6.45%, 9.64%, 41.89%, 9.77%, and 7.18%, respectively. This also means that the developed framework is of great significance for optimizing material composition and improving the performance of materials in civil engineering.
- In this study, the working mechanism of the CatBoost algorithm is explained by using SHAPs and PDPs, and the influence trend and mode of each component of slag–desulfurization gypsum-based alkali-activated materials on the performance are more clearly demonstrated, which provides strong support for the optimal composition of the determined material. This promising tool offers a new horizon for determining the optimal composition of civil materials, promising significant advancements in the field.

## Figures and Tables

**Table 1:** Physical and mechanical properties of slag, fly ash, and Ca(OH)_2_.

Materials	Specific Surface Area/m^2^/kg	Flow Ratio/%	Activity Index/%	Density/g × cm^−3^	Ignition Loss/%	Water Content/%	Bulk Density/g/m^3^	Soakage/%	Stability/%	Fitness/%	
										0.045 mm Material Retained	0.075 mm Material Retained
Slag	429	98	84.2	3.1	0.84	0.45	--	--	--	--	--
Fly ash	0.29	--	--	2.1 × 10^−6^	≤6.5	--	0.79	106	≤4.5	--	--
Ca(OH)_2_	--	--	--	--	22.52	0.23	--	--	--	90.34	99.09

**Table 2:** Chemical composition of slag, fly ash, and Ca(OH)_2_.

Materials	CaO/%	SiO_2_/%	Al_2_O_3_/%	SO_3_/%	Fe_2_O_3_/%	MgO/%	Fe_2_O/%	K_2_O/%	Ca(OH)_2_/%	CaCO_3_/%
Slag	34	34.5	17.7	1.64	1.03	6.01	--	--	--	--
Fly ash	10	40	30	--	--	2.5	4.3	1.2	--	--
Ca(OH)_2_	73.53	0.78	0.597	--	0.128	0.68	--	--	89.51	3.98

**Table 3:** Technical characteristics of desulfurization gypsum and nano-SiO_2_.

Materials	Setting Time (min)		Flexural Strength/MPa	Standard Consistency/%	Expansion Coefficient	Appearance	Density/g/cm^3^	Bulk Density/g/cm^3^	Specific Surface Area/m^2^/g	Particle Size/nm
	Initial Setting	Permanent Setting								
Desulfurization gypsum	3	5	2.5	99	100	White powder	--	--	--	--
Nano-SiO_2_	--	--	--	--	--	White spheres	2.2–2.6	0.06	240	20

**Table 4:** Experimental factors and experimental levels.

Level	CH */%	Fly Ash/%	DG */%	Alkali */%	Modulus *	W/G *
I	2	0	0	2.5	1.00	0.48
II	4	1.75	0.75	3.0	1.05	0.51
III	6	3.50	1.50	3.5	1.10	0.54
IV	8	5.25	2.25	4.0	1.15	0.57
V	10	7.00	3.00	4.5	1.20	0.60

* CH, DG and Alkali represent the content of Ca(OH)_2_, desulfurization gypsum and Na_2_O, respectively. The Modulus is the modulus of Na_2_SiO_3_. W/G represents the water–glue ratio.

**Table 5:** Experimental schemes.

No.	CH/%	Fly Ash/%	DG/%	Alkali/%	Modulus	W/G
1	2	0	0	2.5	1.00	0.48
2	2	1.75	0.75	3	1.05	0.51
3	2	3.5	1.5	3.5	1.10	0.54
4	2	5.25	2.25	4	1.15	0.57
5	2	7	3	4.5	1.20	0.60
6	4	0	0.75	3.5	1.15	0.60
7	4	1.75	1.5	4	1.20	0.48
8	4	3.5	2.25	4.5	1.00	0.51
9	4	5.25	3	2.5	1.05	0.54
10	4	7	0	3	1.10	0.57
11	6	0	1.5	4.5	1.05	0.57
12	6	1.75	2.25	2.5	1.10	0.60
13	6	3.5	3	3	1.15	0.48
14	6	5.25	0	3.5	1.20	0.51
15	6	7	0.75	4	1.00	0.54
16	8	0	2.25	3	1.20	0.54
17	8	1.75	3	3.5	1.00	0.57
18	8	3.5	0	4	1.05	0.60
19	8	5.25	0.75	4.5	1.10	0.48
20	8	7	1.5	2.5	1.15	0.51
21	10	0	3	4	1.10	0.51
22	10	1.75	0	4.5	1.15	0.54
23	10	3.5	0.75	2.5	1.20	0.57
24	10	5.25	1.5	3	1.00	0.60
25	10	7	2.25	3.5	1.05	0.48

**Table 6:** Experimental scales.

No.	Slag/g	NS/g	CH/g	Fly Ash/g	DG/g	Na_2_SiO_3_/g	NaOH/g	Water/g
1	970.2	9.8	20	0	0	50.0	9.7	508.7
2	945.2	9.8	20	17.5	7.5	63.0	12.2	548.4
3	920.2	9.8	20	35	15	77.0	14.9	589.6
4	895.2	9.8	20	52.5	22.5	92.0	17.8	632.6
5	870.2	9.8	20	70	30	108.0	20.9	677.4
6	942.9	9.6	40	0	7.5	80.5	15.6	657.7
7	917.9	9.6	40	17.5	15	96.0	18.6	535.0
8	892.9	9.6	40	35	22.5	90.0	17.4	564.8
9	867.9	9.6	40	52.5	30	52.5	10.2	573.8
10	880.4	9.6	40	70	0	66.0	12.8	614.9
11	915.6	9.4	60	0	15	94.5	18.3	634.3
12	890.6	9.4	60	17.5	22.5	55.0	10.7	639.4
13	865.6	9.4	60	35	30	69.0	13.4	519.5
14	878.1	9.4	60	52.5	0	84.0	16.3	561.1
15	853.1	9.4	60	70	7.5	80.0	15.5	591.6
16	888.3	9.2	80	0	22.5	72.0	14.0	586.4
17	863.3	9.2	80	17.5	30	70.0	13.6	617.6
18	875.8	9.2	80	35	0	84.0	16.3	660.2
19	850.8	9.2	80	52.5	7.5	99.0	19.2	536.7
20	825.8	9.2	80	70	15	57.5	11.1	545.0
21	861	9	100	0	30	88.0	17.1	563.6
22	873.5	9	100	17.5	0	103.5	20.1	606.7
23	848.5	9	100	35	7.5	60.0	11.6	610.8
24	823.5	9	100	52.5	15	60.0	11.6	643.0
25	798.5	9	100	70	22.5	73.5	14.2	522.1

**Table 7:** Test performance of alkali-activated materials.

No.	FS_1d */MPa	FS_3d */MPa	FS_7d */MPa	CS_1d */MPa	CS_3d */MPa	CS_7d */MPa
1	2.1	4.2	7.4	5.5	11.6	15.4
2	2.1	5.4	7.5	4.3	10.8	14.2
3	1.8	5	8.1	3.5	12.4	16.2
4	1.1	3.7	7.4	1	10.6	12.8
5	0.1	3	5.5	0	10.1	13.1
6	2	4.5	8	3.5	8.4	12
7	0.9	6.4	8.7	9.6	17.6	20.6
8	1.5	3.8	8.4	6.6	12.1	16.2
9	1.2	2.8	3.4	1.7	4.5	6.4
10	1.8	4.4	6.2	3.2	8.6	12.3
11	2.2	2.3	5.1	5.3	9.8	14.8
12	1.3	2.4	3.8	1.1	2.7	4.9
13	2.1	4.5	5.8	4.9	8.6	12
14	1.9	5.3	7.6	7.5	12.2	17.3
15	2.1	4.6	6.2	4.7	10.6	14.2
16	1.7	4.3	5.7	3.7	7.8	11.3
17	1.5	2.5	3.5	1.7	3.5	6.7
18	1.6	2.4	3.8	3.9	6.1	10.1
19	2.1	4.8	7.5	10.1	13.2	22.1
20	1.5	4.9	5.1	3.6	6.3	10.1
21	1.8	5.3	6.7	5.1	10.1	12.7
22	1.7	5.1	7.7	7.7	12.6	18.6
23	1.7	3.4	3.9	2.4	5	8.4
24	1.3	2	2.8	1.1	3.3	5.2
25	1.8	5.5	5.6	4.4	8.4	12.1

* FS_1d, FS_3d, FS_7d, CS_1d, CS_3d, and CS_7d represent the flexural strength and compressive strength at 1 day, 3 days, and 7 days, respectively.

**Table 8:** The optimal composition of orthogonal experiments.

Composition	CH/%	Fly Ash/%	DG/%	Alkali/%	Modulus	W/G
Content	2	0	0.75	4.5	1.15	0.48

**Table 9:** The compositions of A1 and A2.

No.	CH/%	Fly Ash/%	DG/%	Alkali/%	Modulus	W/G
A1	12	9.2	4.	3.5	1.15	0.6
A2	12	10	7	4	1.2	0.48

**Table 10:** The experimental and predicted performance of A1 and A2.

No.		CS_1d	CS_3d	CS_7d
A1	Experiment/MPa	3.6	7.03	9.9
	CatBoost/MPa	3.4	7.3	10.7
	Relative error/%	−6.2	3.8	8.1
A2	Experiment/MPa	7.8	13.5	17.1
	CatBoost/MPa	7.2	12.5	16.0
	Relative error/%	−7.69	−7.41	−6.32

**Table 11:** The optimal composition results of SHGO and the orthogonal experiments.

No.	CH/%	Fly Ash/%	DG/%	Alkali/%	Modulus	W/G
Y1	2	0	0.75	4.5	1.15	0.48
Y2	3.1	2.6	0.53	4.3	1.18	0.49

**Table 12:** The results of SHGO and the orthogonal experiments.

Method	No.	FS_1d	FS_3d	FS_7d	CS_1d	CS_3d	CS_7d
CatBoost /MPa	Y1	1.8	5.8	8.2	7.0	14.8	19.4
	Y2	1.1	5.8	8.8	10.2	17.6	21.8
Experiment/MPa	Y1	1.5	6.2	8.3	7.4	17.4	20.9
	Y2	1.9	6.6	9.1	10.5	19.1	22.4

## Data Availability

The data presented in this study are available in the article.
